# Measurements and Models of 915 MHz LoRa Radio Propagation in an Underground Gold Mine

**DOI:** 10.3390/s22228653

**Published:** 2022-11-09

**Authors:** Philip Branch

**Affiliations:** School of Science, Computing and Engineering Technologies, Swinburne University of Technology, Melbourne 3122, Australia; pbranch@swin.edu.au; Tel.: +61-3-9214-5847

**Keywords:** LoRa, sensor networks, RF propagation, underground mining

## Abstract

Underground mining increasingly relies on wireless communications for its operations. The move to automating many underground mining processes makes an understanding of the propagation characteristics of key wireless technologies underground a topic of considerable importance. LoRa has great potential for communications in underground mines, but data on its propagation are quite scarce. In this paper, we describe our measurements of LoRa radio propagation in an underground gold mine. We took measurements in an extraction tunnel with line of sight and in extraction and access tunnels without line of sight. We observed excellent propagation, both with and without line of sight. Our observations support claims by others that the steel-lined tunnels act as a waveguide. As well as reporting measurements, we also developed models of propagation. For line of sight, we show that pathloss is well modelled by a power law with pathloss index of 1.25 and that variability of signal strength is well modelled by a lognormal distribution. We also successfully modelled propagation without line of sight over short distances using a Fresnel Diffraction and Free Space model.

## 1. Introduction

This paper is an extended version of work previously presented at the IEEE International Conference on Information Networking (ICOIN) [1]. This paper recaps the results of that paper but reports in much greater detail the results of non-line-of-sight propagation. The paper’s purpose is to consolidate and provide more detail as to measurements themselves, including details of their analysis not included in the conference paper, as well as report on and summarise additional measurements taken at the boundary of two tunnels without line of sight.

This paper presents measurements and analyses of LoRa propagation in an underground block cave gold mine in New South Wales, Australia. LoRa is an important technology for the Internet of Things with considerable potential for underground mining. However, for it to reach its potential its transmission characteristics underground must be understood. This paper aims to further that understanding.

Block cave mining is a large scale, underground mining technique used for extracting valuable metals, such as gold and copper from large, hard rock, low grade, ore bodies. Block cave mines are very long lasting. A mine might operate for 50 or more years. Block cave mining requires large initial capital costs but is very cost effective over the long life of the mine. Because of their scale, longevity, and the initial capital investment, they are often likened to open pit mines but deep underground.

**Figure 1:**
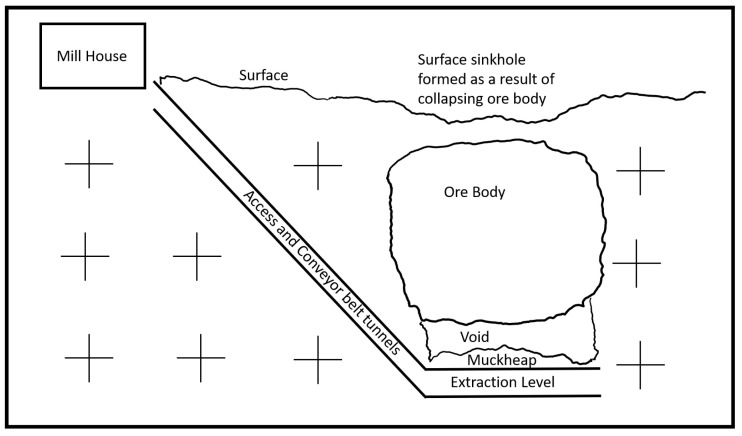
Block cave mine.

**Figure 2:**
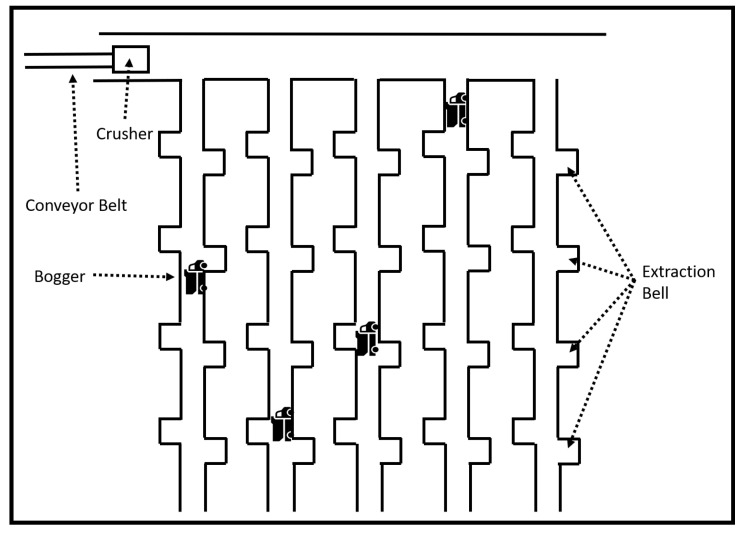
Extraction level.

Development of a block cave mine begins with the construction of an access tunnel (the `main decline’) to underneath the ore body where the extraction level is then constructed. The extraction level is made up of a grid of steel-lined reinforced tunnels (`extraction drives’) with open alcoves (`extraction bells’) from which the ore body is extracted. Above the extraction level, another level is constructed to form the undercut level. Operation begins as the ore body fractures and slowly collapses into the extraction level beneath the ore body where it falls into the extraction bells. From there it is transported via loaders to an underground crusher and then via conveyor belts to the surface. Once delivered to the surface, the ore is milled to extract and refine the minerals. The structure of a block cave mine is shown in Figure 1 while a layout of the extraction level is illustrated in Figure 2.

To ensure the stability of the tunnel and to support the movement of heavy machinery, the walls are lined with steel mesh and then covered in ’spraycrete’ and the floors lined with steel plates.

Data communications play a crucial role in block cave mining in the areas of process control and safety management. Process control underground includes remote control of underground vehicles, crushers, and conveyor belts, as well as detonation of explosives. There is increasing use of autonomous vehicles underground requiring vehicle-to-vehicle (V2V) and vehicle-to-infrastructure communications (V2I) [2]. Safety management requires tracking the location of personnel and vehicles, data from sensors that monitor stability and movement of tunnel walls (“wall deformation”) and environmental data for monitoring for the presence of noxious gases, dust levels, humidity, and temperature.

Underground voice communications is usually VHF radio while data communications is dominated by IEEE 802.11 WiFi with access points connected by an Ethernet backbone. Although there has been some movement towards a possible role for cellular systems, their very high cost generally limits their use underground [3]. There has been some investigation into whether the distributed Radio Access Network of 5G may prove to be more economically feasible than previous cellular generations [4].

With increasing automation and a safety goal of having as few people working underground as possible, the current approach to underground data communications is nearing some limits. Although WiFi is the dominant underground technology, its limited coverage distance is increasingly becoming an issue. For WiFi to have coverage throughout a typical extraction drive 300 m long, directional antenna must be used. WiFi is susceptible to coverage being lost when vehicles in the drive-block line of sight to the access point. For very long tunnels, such as the main decline and conveyor belt shaft, which may be several kilometres long, WiFi access points served by leaky feeder antennae need to be placed every few hundred metres to avoid time-outs. Multiple access points increase maintenance costs and increase communications delay. WiFi also has non-deterministic delay which is an important limitation for industrial process control.

Some of these limitations have led to interest in cellular communications being used underground, notably LTE. LTE has the distance that WiFi lacks, as well as deterministic delay, but its expense usually means it has been deployed only in the main decline and conveyor belt shafts as an alternative to an Ethernet backbone with other technologies deployed in association with it [5].

Consequently, there is increasing interest in the use of Low Power Wide Area Network (LPWAN) technology for communications underground. LPWANs, such as LoRa, have the distance that WiFi lacks yet are much lower cost than LTE. They are also energy efficient and low in maintenance costs. They do not have the bit rate of WiFi and LTE, yet much of the requirements of underground mining are inherently low bit rate. Location data, wall deformation sensors, environmental sensors, crusher actuators and autonomous vehicle communications all require only low bit rates and are a natural fit to LPWAN technology.

Of the LPWANs currently available, LoRa is an excellent data communications match for many underground mining activities. LoRa is low-cost, robust, low in energy demands, has excellent fading characteristics and has a long range of up to 10 or more kilometres [6].

However, for LoRa to be adopted as a communications technology in underground mining it is important to gain an understanding of the propagation characteristics of LoRa underground. In this paper, we report on our measurements of LoRa propagation underground within an extraction drive of a large block cave gold mine. We took measurements of Received Signal Strength Indicator (RSSI) and Signal to Noise Ratio (SNR) for spreading factors of 7, 9, and 12. We took these because the time we had available to take measurements was limited. We did not have time to take measurements for every SF value of 7 to 12 so choose 7, 9, and 12 as representative of low, moderate, and high spreading factors. The values of RSSI are rounded to the nearest whole number. We show that LoRa propagates very well underground, both with and without line of sight perhaps indicative of wave guide behaviour of the steel-lined tunnel. We present models of path loss based on the data for line-of-sight and non-line-of-sight communications. We show that a simple power law can effectively model average path loss, that a lognormal distribution is a good model of RSSI variability at a fixed distance and that Fresnel knife edge diffraction with Free Space Propagation can model RSSI at tunnel junctions.

It is important to note that data on LoRa propagation underground are very rare. We are aware of only one study of LoRa underground and that was not in a mine. The scarcity of data is not surprising because obtaining such data from an operating underground mine is difficult. Measurements need to be taken during periods when the mine is not operating. Gaining access to the mine at such times presents logistical challenges and requires approval from the mine owner whose main priorities are that the mine operates at full capacity and that anyone who ventures underground is safe and does not pose a risk to others. Consequently, such data are very rare and we are grateful to the mine owner and managers for the opportunity to take such measurements.

The paper is structured as follows. Section 2 reviews the literature for data communications underground, as well as providing an overview of LoRa and LoRa-related research. Section 3 describes the theoretical background of this research, as well as how we gathered data for analysis. Section 4 describes and analyses our measurements with line of sight, while Section 5 describes and analyses our measurements without line of sight. Finally, Section 6 is where we summarise this work and point to future research.

## 2. Related Work

### 2.1. Communications for Underground Mining

There has been some research in recent years into wireless communications in underground mining [7,8,9,10,11]. Communications in underground mines are usually based on IEEE 802.11 Access Points connected by an Ethernet backbone [5]. Occasionally, LTE infrastructure is deployed in underground mines [12].

There has been much less research on underground radio signal propagation. The most notable is by Zhou et al., who took many measurements at frequencies ranging from 455 MHz to 5800 MHz [13]. They examined the effect on propagation of polarization and antennae position in mines and tunnels of different types, dimensions, and shapes. They found enormous variation in path loss ranging from as high as 107.79 dB to as low as 1.49 dB per hundred meters. Hakem et al. measured the propagation of 2.4, 5.8, and 60 GHz signals and found that path loss increased as frequency increased [7].

### 2.2. LoRa and LoRaWAN

LoRa and LoRaWAN are increasingly important Internet of Things technologies [14]. LoRa has found widespread use in agriculture, healthcare, renewable energy, industrial processes, and in less obvious applications, such as determining position based on RSSI [15]. Our work has seen it used in underground mining applications of explosive detonation and emergency communications [10,16].

LoRa is a proprietary technology owned by Semtech that defines the physical and datalink layers of the LoRaWAN LPWAN. LoRa’s physical layer is built upon Chirp Spread Spectrum Modulation (CSSM). In LoRa, symbols are modulated onto a rising chirp by using a different starting frequency relative to the lowest frequency of the chirp. LoRa defines a number of different spreading factors (SF) that are mapped to the chirp by using different slopes. The higher the SF the smaller the slope of the chirp [14].

LoRa is power efficient and because it is a spread spectrum technology, very resistant to interference. Distances between transmitter and receiver of up to 15 km have been achieved although more typical values are around one to five km. However, it is low bit rate technology with a maximum rate of 27 kbps although there is a version of LoRa that transmits at 50 kbps but which uses Frequency Shift Keying (FSK) rather than CSSM.

LoRaWAN is a network protocol built on the physical and link layers defined by LoRa [17]. LoRaWAN provides encryption services, a gateway to the Internet, and defines a ’star of stars’ network where each star has, at its centre, a LoRa gateway. The LoRa gateway can provide access to the Internet using protocols, such as MQTT (Message Queue Telemetry Transport) or to other applications via an API, such as RESTful [18]. Contention within LoRaWAN is managed using a simple ALOHA protocol. ALOHA is only effective at low data rates, however for the applications LoRaWAN is mostly used for, low data rates are typical.

Research into LoRa has mostly emphasised its applications. LoRa is particularly well suited to agriculture [19]. Farms where distances are a maximum of a few kilometres match LoRa’s single hop star architecture well. Agriculture applications have included soil moisture monitoring, livestock location and behaviour, and some aquaculture applications. LoRa has been proposed as the basis for an Internet of Medical Things architecture [20]. Medical devices, such as heart rate and blood pressure monitors, would be networked using LoRa. In a similar way LoRa has also been proposed as a means of networking manufacturing process and renewable energy production [21,22].

There has been much less research into LoRa and LoRaWAN itself. There has been some exploration of the scalability of LoRa and LoRaWAN, along with experiments aimed at optimising LoRa parameters to maximise the number of concurrent users [23,24,25,26,27]. There have been some propagation studies aimed at determining how far LoRa can be transmitted successfully [28,29].

LoRa underground has received very little attention. Abrardo and Pozzebon measured LoRa propagation behaviour in mediaeval aqueducts and concluded that they comprised a severe fading environment [30]. The only work on LoRa in underground mining is a simulation study by Emmanual et al. which observed that LoRa is likely to suffer severe fading underground as a consequence of multipath [31]. There appears to have been no actual measurements of LoRa propagation taken in an underground mine.

## 3. Materials and Methods

For all our experiments we used a LoRa transceiver transmitting and receiving at 915 MHz. Our transmit power was 20 dBm.

**Figure 3:**
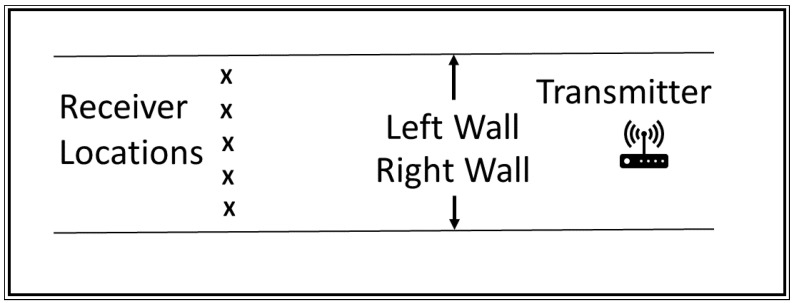
Transmitter and receiver locations (receiver locations shown by "x").

In our line-of-sight experiments, we obtained RSSI and SNR measurements using a LoRa transmitter and receiver using helical antennae. Line of sight measurements were taken in an extraction drive. The dimensions of the drive were approximately 5 m wide and 4 m high. The length of the tunnel was 299 m, however one end of the tunnel was blocked by a steel door and the other occupied by vehicles and machinery, leaving us approximately 240 m of uninterrupted line of sight. We placed the transmitter at a height of approximately two metres at one end of the extraction drive and took measurements every 20 m for a 240 m length of the tunnel. At each 20 m interval we took five measurements across the tunnel at a height of approximately 1.5 m. The five measurements were taken at 1 m and 2 m from the left wall (determined by facing the transmitter), the middle of the tunnel (approximately 2.4 m) and similarly 1 m and 2 m from the right hand wall. Figure 3 shows the location of the transmitter and locations of the receiver. Because of time constraints we took measurements for three different LoRa spreading factors of 7, 9, and 12 rather than for every spreading factor from 7 to 12. As well as recording signal strength, we also recorded the Signal to Noise Ratio (SNR). The other LoRa parameters we used were a bandwidth of 125 kHz and a coding rate of 4. This gave a bit rate of 3.4 kbps at SF 7, 1.1 kbps at SF 9, and 183 bps at SF12. One message was sent every five seconds and the RSSI and SNR measured by the receiver. Each message was 2040 bits in length. We used Dragino LoRa shields connected to Arduino Uno microcontrollers. The transmitter was battery powered and placed on a stand while the receiver was connected to a laptop from which measurements were taken. Measurements were collected on the laptop and later processed using MATLAB. Data is stored on the Swinburne University of Technology data storage system.

We also conducted two sets of experiments, where we measured RSSI and SNR where there was no line of sight. In a block cave mine, extraction drives are connected by an access drive that runs the full length of the extraction level perpendicular to the end point of the grid of extraction drives, as shown in Figure 2. We took measurements with the transmitter located in the access tunnel at distances of 0, 40, 80, and 240 m from the opening of the extraction drive and the receiver located in the extraction drive. For each of these distances we took measurements of RSSI and SNR for the full length of the tunnel.

We considered using ray-tracing to model propagation to determine if we could obtain stronger evidence of a waveguide effect. Unfortunately, the walls of the tunnel are not perfectly straight and the tunnel has some irregularities. There are extraction bells at regular intervals that contain different amounts of ore fallen from the main ore body. To use ray tracing would have required a highly detailed map of the tunnels. Unfortunately, we did not have the time or the permission to construct such a map. Consequently, we are limited in how strongly we can claim to have observed waveguide behaviour. Nevertheless, our results strongly support observations by others that waveguide behaviour in steel-lined tunnels does occur [32].

In the second set of non-line-of-sight experiments we examined signal strength near the junction of an access tunnel and an extraction drive over short distances up to 20 m.

In Section 4, we discuss line-of-sight measurements and in Section 5 both sets of non-line-of-sight measurements.

## 4. Path Loss with Line of Sight

Theoretical considerations as described in [33] suggests that average signal strength $P_{d}$ can be modelled as a power law against distance *d* as follows:

(1) $$P_{d}=P_{d_{0}}\frac{d_{0}}{d^{n}}$$

where $P_{d}$ is the mean power at distance *d* and $P_{d_{0}}$ is the mean power at a reference distance (usually a distance of one unit from the transmitter) of $d_{0}$. This equation is called the “close in equation”. This is usually expressed in dB:

(2) $$P_{d}\left(dB\right)=P_{d_{0}}\left(dB\right)-10n\log_{10}\left(d\right)$$

For a power law, Equation (2) is a straight line with negative slope of *n* when plotted against log of distance. The path loss exponent *n* is the key variable in developing a model of path loss. It has typical values between 2 and 4. A larger value of *n* indicates a more severe path loss environment. Determining *n* enables us to model the mean value RSSI at specific distances. However, we also need to know the distribution of RSSI at a fixed distance. Experimental observations and some theoretical results suggest that path loss at a fixed distance is well modelled by a lognormal distribution or a normal distribution in dB [33]. In the following sections, we demonstrate that signal strength underground with line of sight matches these models well.

### 4.1. Path Loss Exponent

As noted, the key variable in modelling path loss is the path loss exponent *n*. The pathloss exponent is the slope of the line when RSSI in dB is plotted against decades of distance where a decade is when the distance between the transmitter and the receiver increases by a factor of 10.

**Figure 4:**
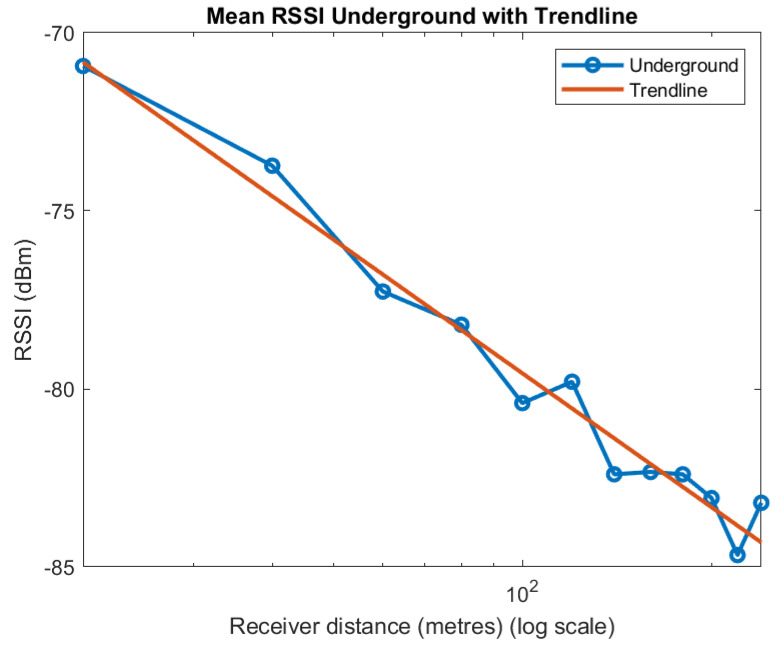
RSSI and trendline.

We determine *n* by taking the average of all measurements at distances of 20 to 240 m in 20 m increments. As noted we took a total of 15 measurements at each distance made up of 5 measurements taken across the tunnel for each of the three spreading factors we used of 7, 9, and 12. Figure 4 shows the mean value of RSSI against decades of distance. It is worth noting the good fit to a straight line of the plot. The slope of this line is −1.25. That is, the Path Loss exponent is 1.25. Free space has a Path Loss exponent of 2, indicating that loss over long distances is less underground than in free space.

### 4.2. Distribution of RSSI Variation

Because of constructive and destructive interference caused by multipath, RSSI will vary at the same distance in different locations across the drive. On the surface this is referred to as `shadow’ or `slow’ fading. Shadow fading on the surface is usually modelled with a lognormal distribution. In this section we look at the variability of RSSI across the tunnel at each distance. For our measurements the variation is indeed well described by a lognormal distribution with a standard deviation of 4.5 dB.

**Figure 5:**
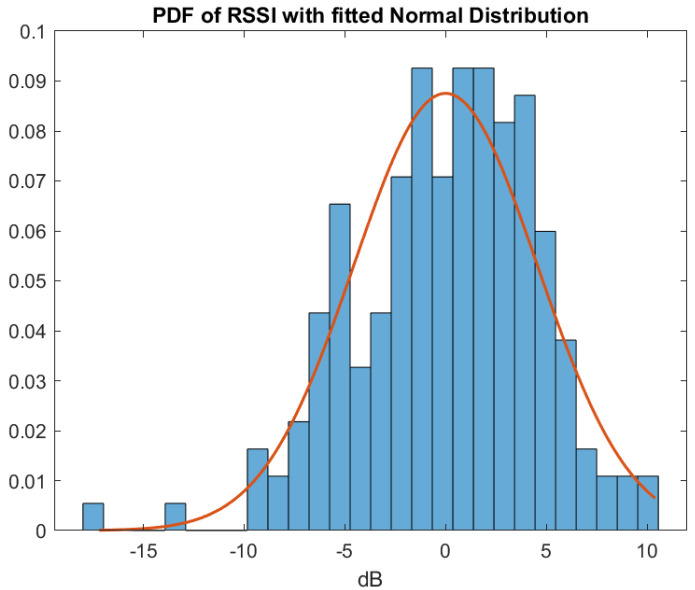
Histogram of RSSI variation from mean. (Observations are blue. Fitted Normal Curve is red).

Figure 5 shows a histogram of the variation from the mean of all our samples. These values were obtained by calculating the mean RSSI of the 15 measurements at each distance and then subtracting the mean from each sample giving a total of 180 samples. The histogram is overlaid with a normal distribution with the sample variance calculated from the measurements. We see that there is a good match between the distribution of the samples and the derived distribution.

The other diagram used to explore the distribution of the variation at a fixed distance is a Quantile–Quantile (Q–Q) plot. Q–Q plots give a visual representation as to how well a set of samples matches a distribution derived from the data. A Q–Q plot is created by plotting the set of quantiles from the sample distribution against the set of quantiles from the derived distribution. The nearer the points are to a straight line the better the match. More formally, a Q–Q plot is a scatter plot where each pair of values comprises a quantile from the derived distribution and the observed distribution. Quantiles are usually equal to the number of sample points. In our case, the mean and variance of a normal distribution is estimated from the samples. The quantiles of the sample distribution are plotted against the corresponding quantiles of the collected samples.

**Figure 6:**
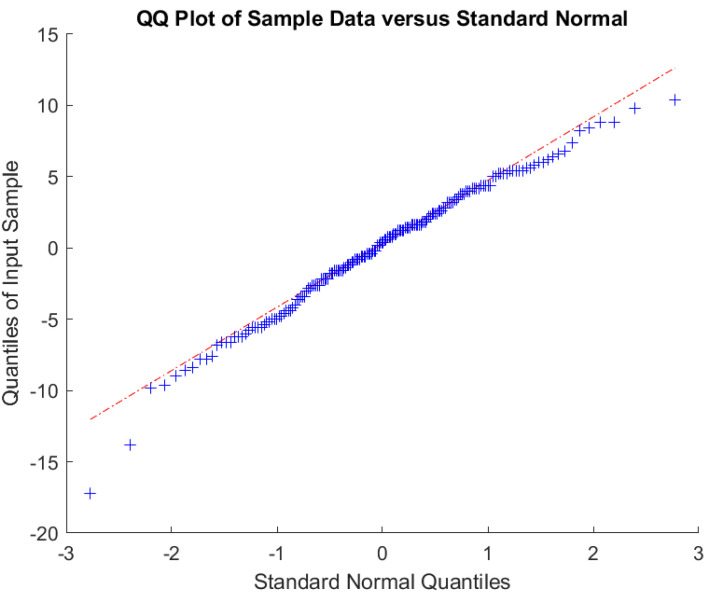
Q–Q plot of variation and normal distribution.

In Figure 6, the data are plotted against a derived normal distribution with the same mean and variance.

Once again, we see a good match between the derived distribution and the sample distribution.

Finally, we can use the Shapiro–Wilk test to see whether there is evidence to reject the null hypothesis that the sample values have a normal distribution [34]. A *p* value greater than 0.05 means there is no evidence to reject the hypothesis that the data are normally distributed. Our *p* value for the sample data when tested for being normal is 0.0528, meaning that the data does not differ significantly from a normal distribution.

Consequently, we can claim that RSSI variability within the extraction drive can be well modelled with a lognormal distribution with standard deviation of 4.5 dB.

### 4.3. Effect of Spreading Factor on RSSI

**Figure 7:**
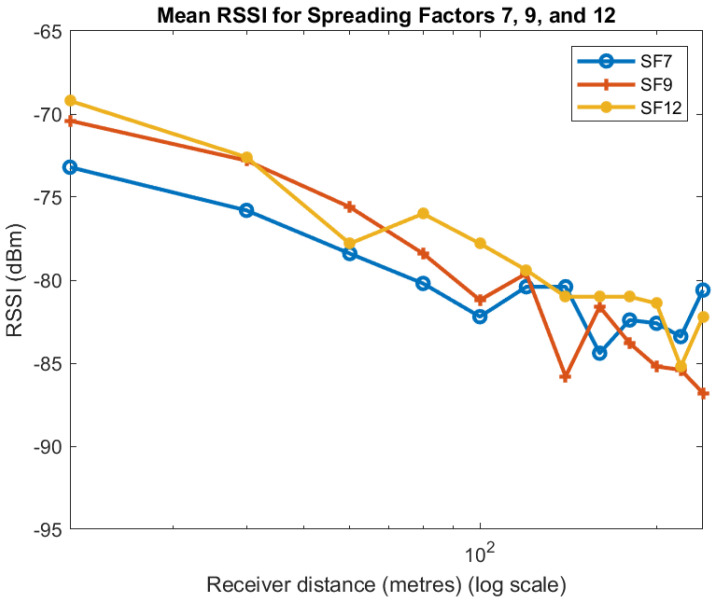
Effect of SF on RSSI.

We now look at the data to determine what effect the spreading factor (SF) has on RSSI. The data for the three SFs are plotted in Figure 7. From the plots, we see that for distances up to 120 m a higher spreading factor mostly gives a higher RSSI. However, for distances beyond 120 m, there is no discernable pattern.

### 4.4. Effect of Position within Tunnel on RSSI

We now analyse the impact that position within the tunnel has on RSSI. For this analysis measurements are classified into two categories: near the wall and near the centre.

**Figure 8:**
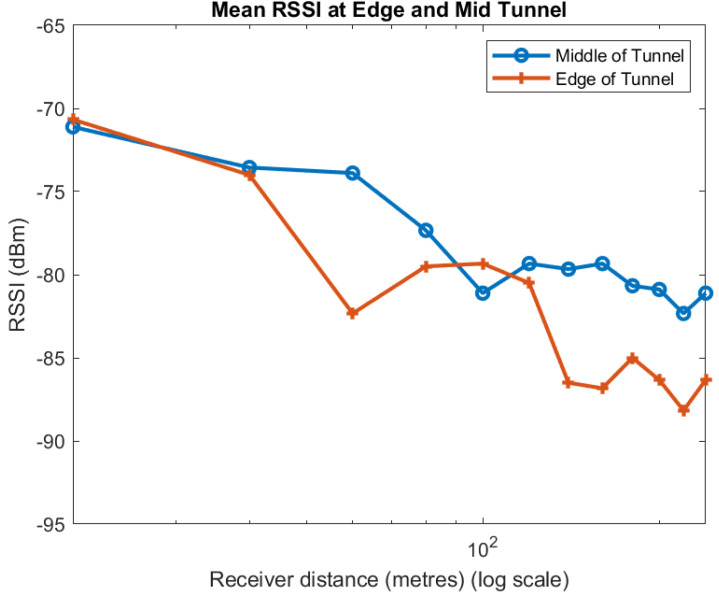
Effect of position in tunnel on RSSI: mean for all SF.

At each distance, we took five measurements across the tunnel at 1 m and 2 m from each wall and in the centre of the tunnel 2.4 m from both walls. We categorise the two measurements 1 m from both walls as being near the wall and the two measurements 2 m from either wall and in the centre of the tunnel as being near the centre. We then take the mean of each category at each distance. Figure 8 shows the mean across all spreading factors of the RSSI in the middle and sides of the tunnel.

**Figure 9:**
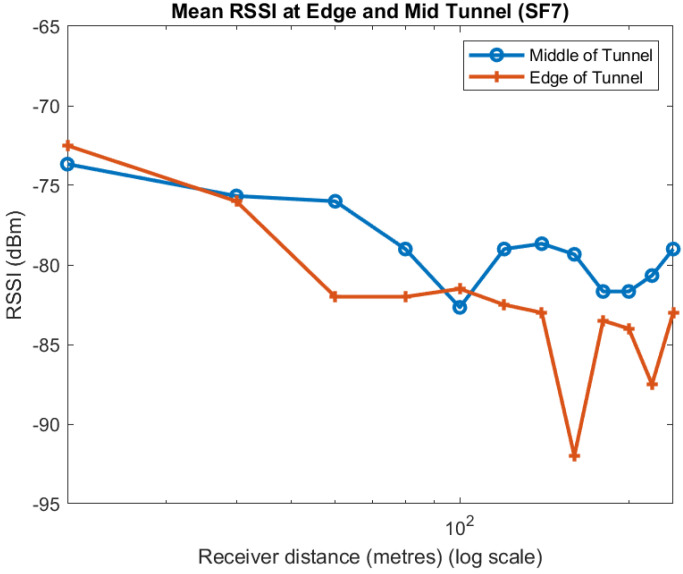
Effect of position in tunnel on RSSI: SF7.

**Figure 10:**
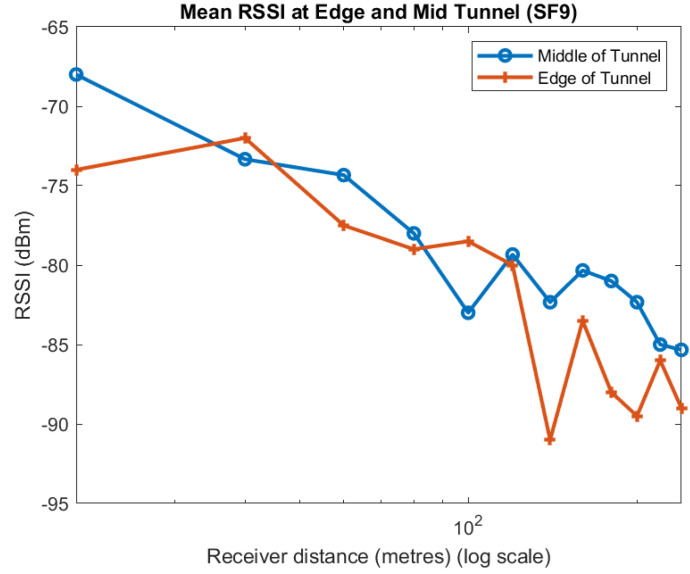
Effect of position in tunnel on RSSI: SF9.

**Figure 11:**
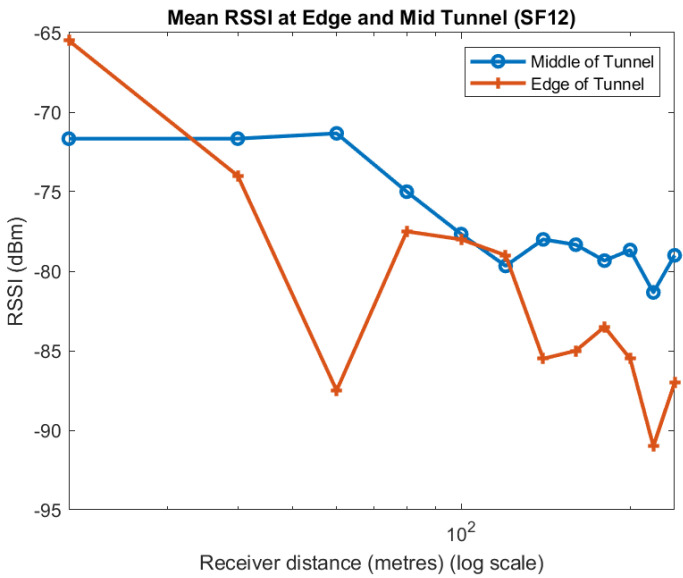
Effect of position in tunnel on RSSI: SF12.

Apart from one point at 100 m, the mean of the measurements taken near the centre of the tunnel always have a higher RSSI than near the walls. We observe much the same for the individual spreading factors, as shown in Figure 9, Figure 10 and Figure 11. Although the trend in the data is consistent, we note that there is quite a lot of variability in the different plots. A likely explanation is that scattering off the rough surfaces and bulges of the tunnel is a significant mode of propagation.

### 4.5. Signal to Noise Ratio

The SNR was quite consistent for SF 7 and SF 9. For SF 7 the SNR was 9 dB and for SF 9 the SNR increased to 13 dB. Time constraints meant we did not take measurements of SNR for SF 12. We now reconcile these observations with theoretical modelling of spread spectrum communications. We observed an increase in SNR of 4 dB in going from SF 7 (SNR of 9 dB) to SF 9 (13 dB).

In a spread spectrum system, the “Processing Gain” is a measurement of the gain in signal strength when the message signal is despread. This value, *G*, is the bandwidth divided by the bit rate. That is G=BW/bitrate. Consequently, the difference between signal gains at SF 7 $G_{7}$ and at SF 9 $G_{9}$ should differ by a factor equal to the ratio of the bit rates at each SF. The bit rate at SF7 is 5.47 kbps and at SF 9 is 1.76 kbps. Consequently we should see an increase in the SNR when going from SF 7 to SF 9 equal to 5.47 kbps/1.76 kbps which is 3.1. Expressed in dB, this is 4.9 dB, which is in good agreement with our observed increase of 4 dB.

## 5. LoRa Propagation without Line of Sight

To determine the propagation characteristics without line of sight, we conducted two sets of experiments.

In the first set of experiments, we placed the transmitter at 0, 40, 80, and 240 m from the junction in the centre of the access tunnel and took measurements every 20 m from 20 m to 200 m along the length of the centre of the perpendicular extraction tunnel.

**Figure 12:**
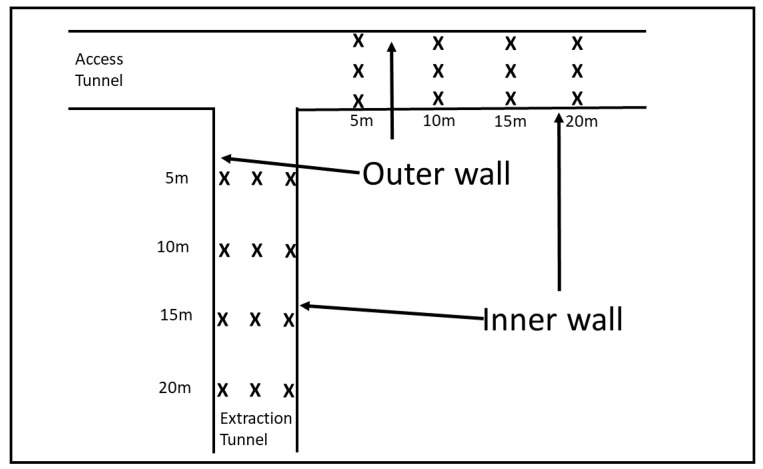
Tunnel junction locations.

In the second set of experiments, we were interested in short range effects near the junction of the two tunnels. To explore this we took measurements of RSSI at a junction between the access tunnel and the extraction tunnel. The locations of the measurements are shown in Figure 12. The transmitter was placed at each location in the access tunnel marked with an ’X’ while the receiver was placed at each location in the extraction tunnel also marked with an ’X’.

The transmitter and the receiver were placed at 5, 10, 15, and 20 m from the junction of the tunnel at 3 locations across the tunnel. The three locations across the tunnel were 1 m from the rightmost wall looking towards the junction, midway between the walls, and 1 m from the leftmost wall looking towards the junction. The tunnels were approximately 5 m in width and 4 m in height.

Measurements were taken for every pair of transmit and receiver locations for SF of 7 and 9 giving a total of 288 measurements. In this paper, we report on measurements taken with the receiver placed along the inner wall and the transmitter also placed along the inner wall, the receiver placed in the mid-tunnel and the transmitter placed mid-tunnel, and of the receiver placed along the outer wall and the transmitter placed along the outerwall corresponding to a total of 96 individual measurements. The full dataset is available upon request.

We present the results of each set of experiments in the next two subsections.

### 5.1. Measurements along the Length of the Extraction Tunnel

**Figure 13:**
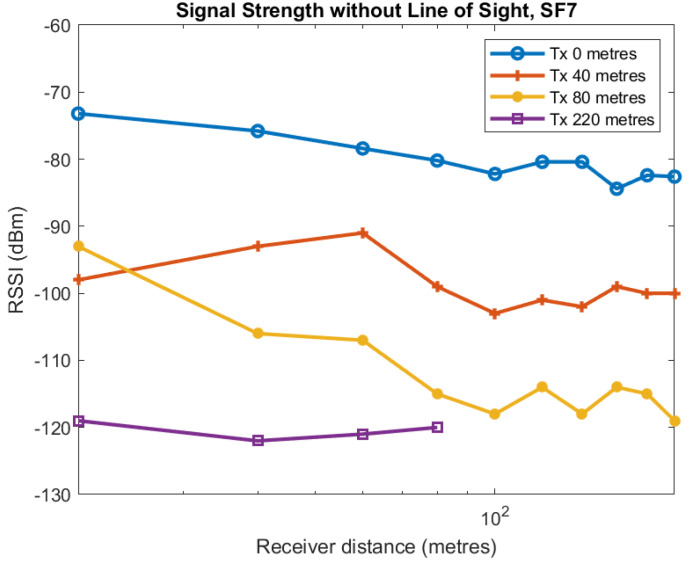
RSSI against log of Receiver distance from junction for different transmitter distances from junction.

**Figure 14:**
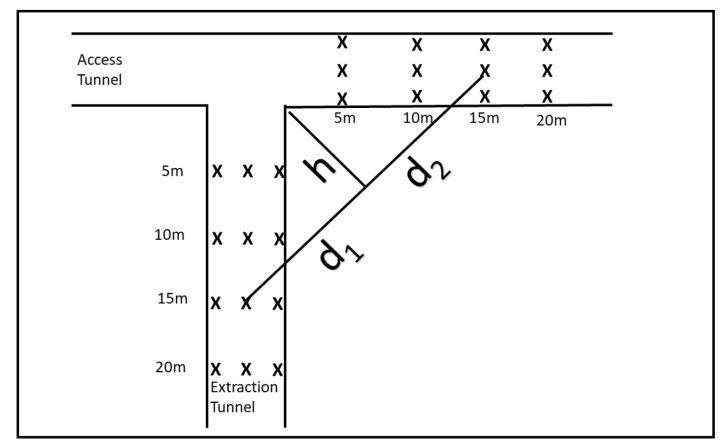
Knife edge diffraction modelling. Transmitter and Receiver locations shown by "x".

In this section, we describe measurements taken with the transmitter and receiver both in the centre of the tunnel. These measurements are shown in Figure 13. The purpose of this work was to see how far the signal would propagate without line of sight, as well as understand how the signal strength at the receiver changed when both the transmitter and receiver were substantial distances from the junction. The results demonstrate that even without line of sight the signal propagates an impressive distance. Figure 13 comprises four lines. Measurements represented by the first line were taken with the transmitter right at the junction of the two tunnels in the middle of the access tunnel perpendicular to the extraction tunnel (see Figure 14 for relative position of the access and extraction tunnels). The first line shows that RSSI drops quite smoothly and slowly. When the transmitter is placed 40 m within the access tunnel the RSSI measured in the extraction tunnel varies quite substantially between the range of −90 and −100 dBm. When the transmitter is placed 80 m from the junction within the access tunnel measurements taken in the extraction tunnel drop more severely. Finally, we placed the transmitter the full 240 m at the end of the access tunnel and took measurements in the extraction tunnel. Interestingly, they stayed reasonably constant until at 60 m they dropped suddenly below the detection level of the receiver. We took no further measurements beyond that distance. LoRa has a sensitivity of around −130 dBm. Since the signal was around −120 dBm for all measurements it is not surprising it did not reach the full length of the tunnel. Nevertheless, it is impressive just how far the signal propagates without line of sight.

The key point to take from this subsection is that LoRa propagates well within these steel lined tunnels, even without line of sight.

### 5.2. Measurements near the Tunnel Junction

The final set of measurements was taken to explore signal strength over a short distance without line of sight at a tunnel junction. We were particularly interested in seeing if the Fresnel knife-edge diffraction model could predict signal loss over distances of up to 20 m from the junction.

We placed the transmitter at 5, 10, 15, and 20 m from the tunnel junction. At each distance the transmitter was located at three locations across the tunnel. This resulted in 12 different transmitter locations. For each transmitter location we took measurements at the receiver at the same locations in the perpendicular tunnel also at 5, 10, 15, and 20 m from the junction of the tunnels. At each distance the receiver was placed in three locations across the tunnel giving a total of 12 receiver locations, as shown in Figure 12. For each combination of receiver and transmitter location we took signal strength measurements for SF of both 7 and 9 giving a total of 288 measurements of signal strength near the tunnel junction. The locations of the transmitter and receiver are shown in Figure 12. This diagram defines what we mean by inner and outer wall which are terms used below.

From the results, we see that signal strength decreases the nearer the receiver or transmitter is to the inner wall. We also see quite a substantial drop in signal strength as the distance between the transmitter and receiver increases.

**Figure 15:**
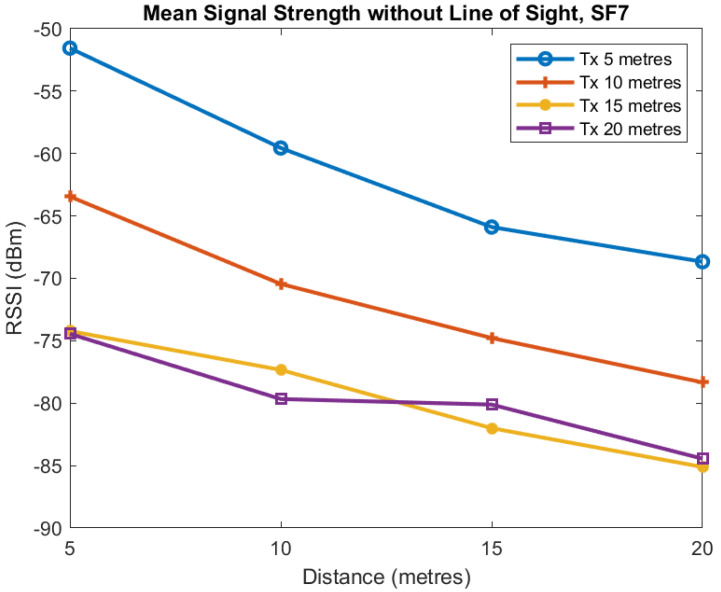
Mean RSSI against receiver distance from junction for different transmitter distances from junction, SF7.

**Figure 16:**
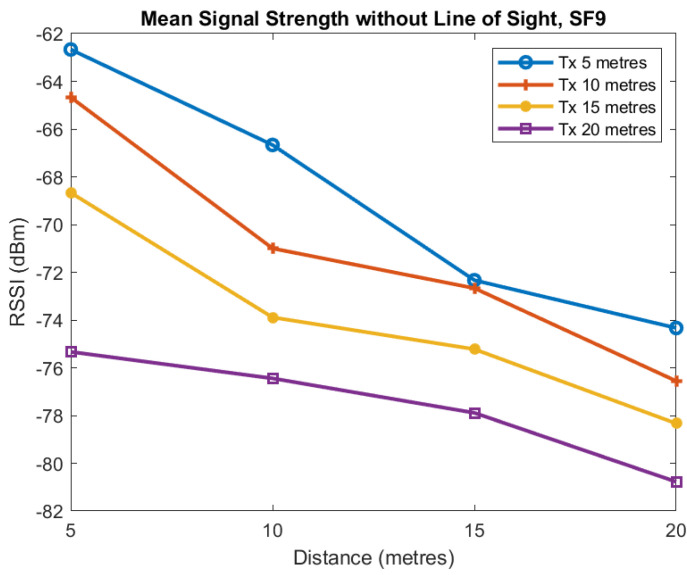
Mean RSSI against receiver distance from junction for different transmitter distances from junction, SF9.

The first two plots show the mean signal strength for each distance for SF7 and SF9. Each of the four lines in Figure 15 and Figure 16 is for the transmitter located at 5, 10, 15, and 20 m from the tunnel junction with the horizontal axis being the distance of the receiver from the junction. The vertical axis shows the RSSI in dBm measured at the receiver.

These plots of average value show reasonably smooth behaviour as the distance increases although there is some unusual behaviour when the transmitter is at distance of 20 m and Spreading Factor is 7. For SF7 increasing the transmitter distance from the tunnel from 5 to 10 m causes approximately a 10 dB loss and a similar loss when the transmitter distance is increased from 10 to 15 m. However, when the transmitter distance is increased to 20 m we see no additional loss. For SF9, we see similar behaviour as distance is increased although the loss is less but more variable.

**Figure 17:**
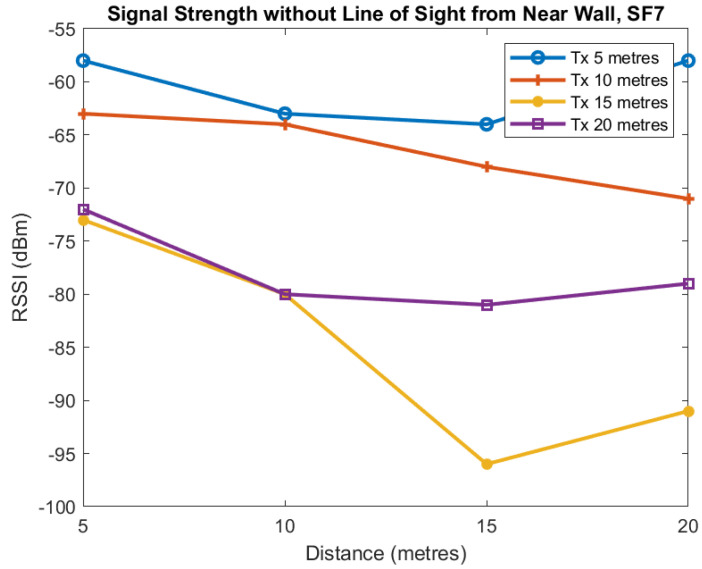
RSSI against receiver distance from junction for different transmitter distances from junction, Near-Wall, SF7.

**Figure 18:**
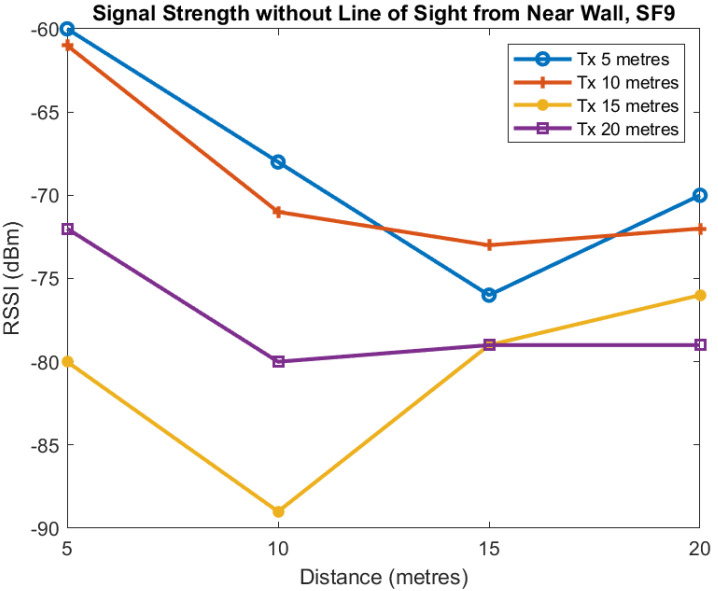
RSSI against receiver distance from junction for different transmitter distances from junction, Near-Wall, SF9.

We now present data in Figure 17 and Figure 18 for the transmitter and receiver both located at the inner wall. We present two plots: one for SF7 and the other for SF9. Access to the mine and time constraints meant we were unable to take additional measurements. The inner and outer walls referred to in this section are shown in Figure 12. Each line corresponds to a transmitter located at the inner wall 5, 10, 15, and 20 m from the junction with the horizontal axis being the distance of the receiver from the junction. The vertical axis shows the RSSI in dBm measured at the receiver. We see a general trend of the RSSI decreasing as the distance of the transmitter and receiver from the junction is increased but there is a great deal of variability.

**Figure 19:**
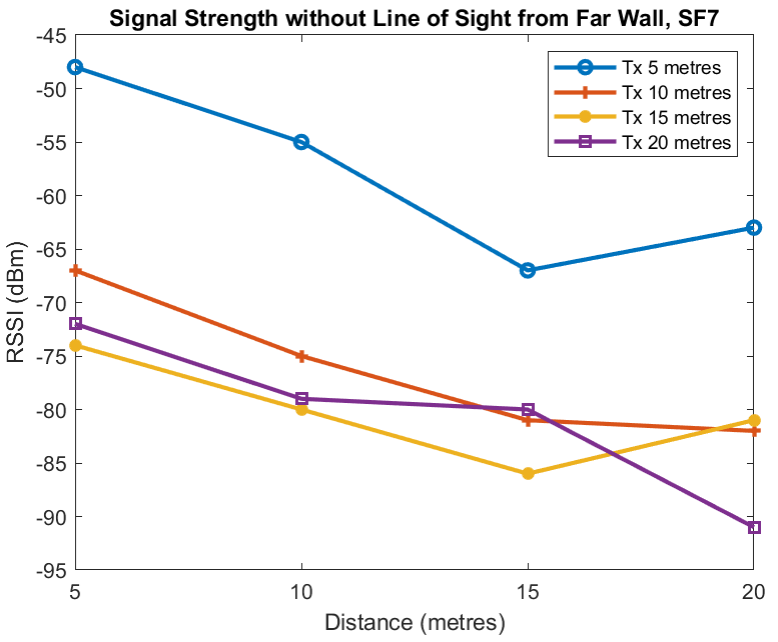
RSSI against receiver distance from junction for different transmitter distances from junction, Far-Wall, SF7.

**Figure 20:**
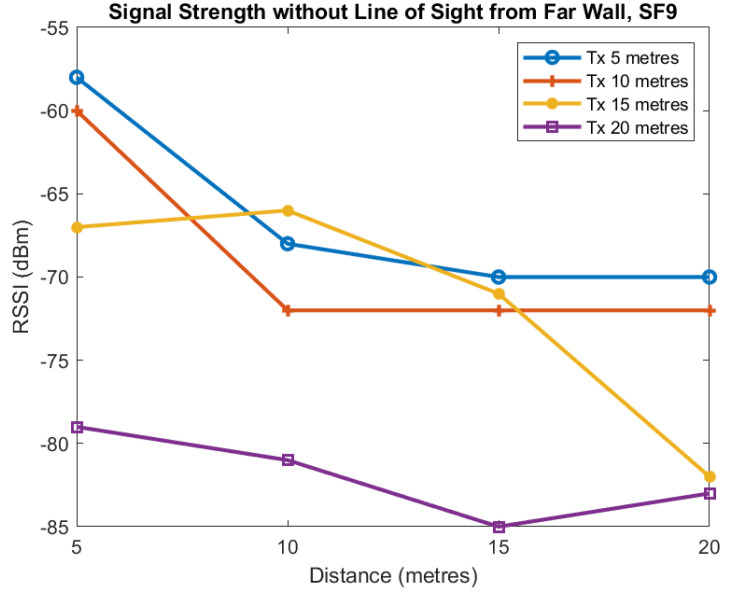
RSSI against receiver distance from junction for different transmitter distances from junction, Far-Wall, SF9.

Finally we present data for the transmitter and receiver both located at the outer wall for SF7 (Figure 19) and SF9 (Figure 20). As before, we see a general trend of the RSSI decreasing as the distance the transmitter and receiver from the junction is increased. In this case, there is some variability, but not as much as seen for the inner wall.

### 5.3. Diffraction Modelling of Signal Strength at Tunnel Junction

In this subsection, we demonstrate that Fresnel Knife Edge Diffraction Model in free space is an acceptable model for signal propagation at the short distances near the tunnel junction [33]. The Fresnel Knife Edge Diffraction Model is dependent on the Fresnel Diffraction Parameter ν defined in terms of the distance to the knife edge of the transmitter $d_{1}$, the distance from the knife edge to the receiver $d_{2}$, the wavelength of the signal λ, and the height of the knife edge obstruction *h*. Figure 14 shows the relationship between $d_{1}$, $d_{2}$, and *h* where the transmitter and receiver are both located in the centre of the tunnel and are both 15 m from the tunnel junction. The model assumes that there is no signal propagated through the knife edge and that propagation between the transmitter and the knife edge, and the knife edge and the receiver is modelled by free space. The parameter ν is defined as:

(3) $$\nu =h\sqrt{\frac{2(d_{1}+d_{2})}{\left(\lambda d_{1}d_{2}\right)}}$$

Using ν the diffraction loss G(ν) can be estimated by:

(4) $$G\left(\nu \right)=6.9+20log_{10}\left(\sqrt{{\left(\nu -0.1\right)}^{2}+1}+\nu -0.1\right)$$

Loss in free space is predicted by the following equation where $G_{t}$ and $G_{r}$ are the transmitter and receiver antennae gains while λ is the wavelength and *d* is the distance between the transmitter and receiver.

(5) $$PL\left(dB\right)=G_{t}+G_{r}+20log_{10}\left(\frac{\lambda }{4\pi d}\right)$$

To illustrate the process of predicting path loss due to diffraction and freespace we show the calculations involved with the transmitter and receiver both 10 m from the junction and 1 m from the inner wall. For determining loss due to free space, we need to determine the distance *d* in Equation (5). The distance to the knife edge is 10 m and from the knife edge to the receiver is another 10 m. The antenna gains are both 1 dBi. This corresponds to a total distance *d* = 20 m. Using Equation (5), above, with frequency 915 MHz, we obtain loss in free space of 53 dB. To calculate the loss due to diffraction, we note from Figure 14 that *h*, $d_{1}$, and $d_{2}$ are all approximately 7 giving us a ν value of 9.16. Then, using Equation (4) we obtain a diffraction loss of 32 dB. The total loss due to free space and diffraction is then 85 dB. To compare this prediction with observation we note that the transmitter power is 20 dBm while the RSSI value observed at the receiver is −64 dBm corresponding to a loss of 84 dB which compares well with the predicted loss.

**Figure 21:**
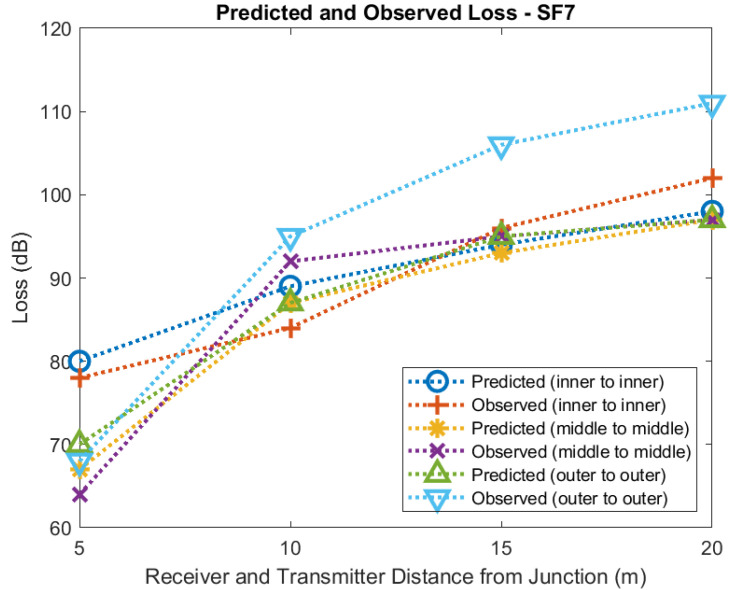
Diffraction modelling of predicted and observed loss at tunnel junction (SF7).

**Figure 22:**
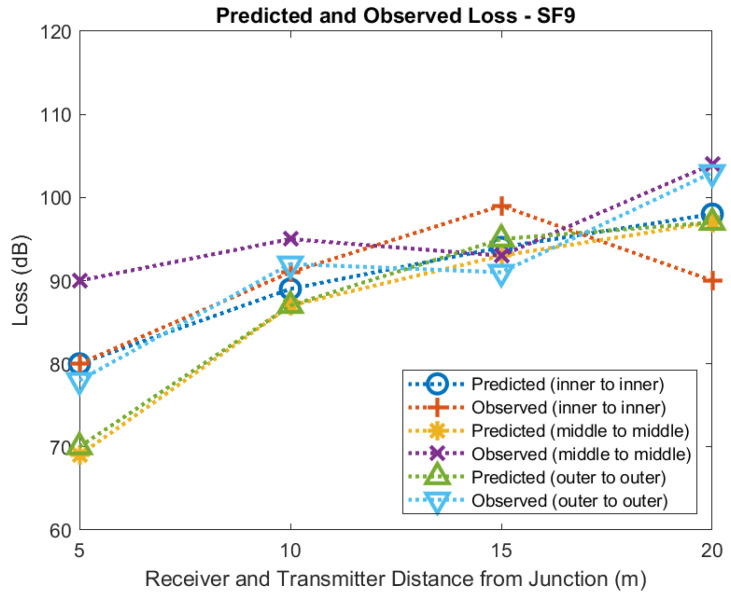
Diffraction modelling of predicted and observed loss at tunnel junction (SF9).

Figure 21 and Figure 22 show predicted and observed path loss for the transmitter and receiver equal distances from the tunnel junction for SF7 and SF9, respectively. Each plot shows loss for measurements taken with the receiver and transmitter against the inner wall, the receiver and transmitter in the middle of the tunnel, and for the receiver and transmitter placed against the outer wall. Agreement between predicted and observed values is quite good for measurements taken against the inner wall and in the middle of the tunnel but is poorer for measurements taken against the outer tunnel. Additionally, agreement between observed and predicted for SF9 is quite good but not so good for SF7.

It may be that for the middle of the tunnel and outer wall measurements other mechanisms, perhaps reflection, have a significant effect on propagation. Nevertheless, our model and measurements suggest that Fresnel Knife Edge Diffraction in Free Space is able to provide a reasonable approximation to path loss over these short distances.

## 6. Conclusions and Further Work

This paper presents models based on data of the propagation of LoRa in an underground goldmine based on data collected over a period of a few days in an operational goldmine. We demonstrate that a log distance model with pathloss exponent of 1.25 models the mean signal strength against distance and a lognormal distribution with mean 4.5 dB models signal strength variability. We also demonstrate that short distances without line of sight near tunnel junctions are well modelled by Fresnel Diffraction with path loss of 2. We also present considerable amounts of data showing the effect of spreading factor on signal strength. We showed that SF has a variable impact on RSSI and a more consistent impact on SNR. RSSI was mostly greater with larger SF. We also observed an increase in SNR of 4 dB between SF of 7 and 9.

Our results suggest LoRa propagates well in a block cave mine. Our results differ markedly from the very little other research into LoRa propagation underground which found it propagated poorly. We attribute the difference to waveguide behaviour caused by wire mesh on the wall and ceiling, and steel plates on the floor of the tunnels. Waveguide behaviour has previously been observed in underground mines lined with steel.

We also report on signal propagation without line of sight. We demonstrated that even without line of sight the signal propagated a substantial distance in a perpendicular tunnel. We also measured signal strength without line of sight over short distances at a tunnel junction.

The research described in this paper gives us a good understanding of how LoRa propagates in an underground block cave mine. We intend using that understanding to develop mining applications of LoRa underground. We have already prototyped two systems: one for explosives detonation [10] and the other for location communications underground [16]. We are keen to develop these further so as to meet the very high safety standards for underground mining, as well as develop other applications of LoRa for underground mining.

LoRa is an important IoT technology with great potential for underground mining. For its potential to be realised, an understanding of how it propagates underground is needed. This paper makes a contribution to that understanding.

## Data Availability

Data will be available in a publicly available repository. Contact the author for details.
